# Bibliometric Evaluation of 2012–2020 Publications on Ferroptosis in Cancer Treatment

**DOI:** 10.3389/fcell.2021.793347

**Published:** 2022-01-18

**Authors:** Qian Zhou, Fei Wu, Mingyi Zhao, Minghua Yang

**Affiliations:** ^1^ Department of Pediatrics, The Third Xiangya Hospital, Central South University, Changsha, China; ^2^ Xiangya School of Medicine, Central South University, Changsha, China

**Keywords:** ferroptosis, cancer treatment, bibliometric analysis, citespace, VOSviewer, visualization analysis

## Abstract

Ferroptosis is an iron-dependent regulated cell-death caused by the accumulation of lipid peroxides, which is different from apoptosis, autophagy, necroptosis and other forms of regulatory cell death in morphology and mechanism. It has attracted interest and attention of scholars from all over the world during the past years. Recent studies have shown that ferroptosis is able to play a clear and important role in cancer treatment, providing a bright prospect for targeted cancer therapy. This article aims to analyze current scientific results about the application of ferroptosis in the field of cancer treatment, providing new ideas for further research. We retrieved publications related to ferroptosis and cancer treatment from 2012 to 2020 from the Web of Science Core Collection (WoSCC), screening them according to the inclusion criteria. A total of 965 related papers were included, and the total number of publications increased year by year. We used CiteSpace 5.7. R2, VOSviewer and Microsoft Excel 2019 to evaluate and visualize the results, analyzing institutions, countries/regions, journals, authors, co-cited reference and keywords. Overall, with more and more evidence showing the indispensable role of ferroptosis in cancer, its mechanism research and target discovery may become the main direction of future research.

## Introduction

Ferroptosis is a specific iron-dependent regulated cell-death associated with lipid peroxidation, primarily driven by the accumulation of lipid hydroperoxides (Yang et al., 2014; Cao and Dixon, 2016; Yang and Stockwell, 2016). Morphologically, it is characterized by high density of mitochondrial cell membrane, reduction or disappearance of mitochondrial cristae, rupture of mitochondrial outer membrane and other cytological changes, which was first proposed by Dixon et al. (2012), Xie et al. (2016).

Ferroptosis plays an important role in a variety of diseases, such as cancer (Angeli et al., 2014; Shen et al., 2018; Wu et al., 2020), kidney failure (Müller et al., 2017; Deng et al., 2019; Weiland et al., 2019), brain disease (Fang et al., 2019; Kenny et al., 2019; Nagpal et al., 2019), cardiovascular disease (Wang et al., 2017; Del Re et al., 2019; Capelletti et al., 2020), liver disease (Holohan et al., 2013; Zhang et al., 2019; Mao et al., 2020) has been gradually identified during these years. Among them, cancer research is undoubtedly a hot spot due to its high risk and mortality. Experts all over the world have been struggling to develop more effective and safer drugs to fight against cancer. Current anti-cancer drugs kill cancer cells mainly by inducing apoptosis. However, resistance to anti-cancer drugs limits the effectiveness of current cancer therapies and is a major problem in present-day cancer research (Sun et al., 2016; Cao, 2019). Therefore, it is urgent and meaningful to search for and develop new cancer drugs. Ferroptosis, a form of non-apoptotic cell death, can serve as a novel pathway for cancer treatment.

There are two main mechanisms of ferroptosis against cancer. The most important one is to inhibit the glutamate-cystine antiporter system (System Xc-), which reduces the uptake of GSH, leading to lipid peroxidation and ferroptosis. It can be achieved by erastin. In addition, RSL3 and DPI7 can directly inhibit the activity of GPX4 to induce ferroptosis, thus exerting a therapeutic effect on cancer (Yang et al., 2014; Hangauer et al., 2017). Meanwhile, Vasanthi SV *et al.* proposed that the survival of some drug-resistant cancer cells depends on the survival of GPX4, which makes GPX4 an ideal target of ferroptosis to kill cancer cells, so as to prevent and cure cancer recurrence (Wang et al., 2017; Eaton et al., 2020). Thus, the development of effective GPX4 covalent inhibitors, such as ML210, has gradually aroused general interest in anti-cancer drug research (Wang et al., 2019). However, few systematic analyses have been performed over the past years. Studies have shown that cancer immunotherapy can enhance the sensitivity of tumors to traditional radiotherapy by promoting ferroptosis. Specifically, CD8^+^ T cells down-regulate SLC3A2 and SLC7A11 (two key units of the system Xc-) by releasing INF to induce ferroptosis, which may be one of the effective methods to reduce the tolerance of cancer treatment (Ubellacker et al., 2020). In the same way, other ferroptosis inducers can also play a role in inhibiting tumors. Therefore, the exploration of mechanisms is essential for further research and now is the focus of study in this field. Ferroptosis is a biological process regulated by multiple genes, accompanied by a series of morphological and metabolic changes. Consequently, through in-depth research on the physiology, cytology, molecular biology, genomics and bioinformatics of ferroptosis, more cancer therapeutic targets may be discovered.

Bibliometric analysis is a comprehensive knowledge system focusing on quantification, and has been widely used to gain insight into scientific development trends and provide researchers with a reference when choosing the research direction. As far as we know, there is currently no bibliometric analysis on cancer research of ferroptosis, however, there have been three bibliometric analyses articles in the field of ferroptosis (Wu et al., 2021; Xiong et al., 2021; Zhang et al., 2021), which have a great reference for our research. Therefore, we decided to carry a bibliometric analysis in this area, especifically from the perspectives of countries, journals, authors and keywords to analyze the trends and hotspots of cancer research of ferroptosis, in order to provide guidance for scholars in this area to determine the research direction.

## Methods

### Search Strategies

We searched publications on cancer research of ferroptosis in January 2021 by using the Science Citation Index Expanded database of the Web of Science Core Collection (WoSCC). The search terms that we used were as follows: ferroptosis AND (cancer OR tumor OR neoplasm OR carcinoma OR adenocarcinoma OR leukemia OR leukemia OR sarcoma OR lymphoma OR oncology). Only publications before 2021 were included. A total of 965 documents met the search criteria, which were applied in this study.

### Data Collection and Analysis

We exported the full record of data from WoSCC, including the number of annual publications; outputs of countries/regions, journals, authors and total citations; impact factor (IF) in 2019, Journal Citation Reports (JCR) 2019 and Hirsch index (H-index). JCR is a database which serves as the basis of Journal Impact Factor (JIF), which is a journal evaluation tool that can reflect the impact and quality of journals. And H-index means that H papers published by an author or a country/region have been cited at least H times, and each of the remaining papers has been cited less than or equal to H times. It can be used to evaluate the amount and level of academic output of researchers or countries/regions.

Then, we imported the data to Microsoft Excel 2019, VOSviewer and CiteSpace 5.7. R2 for further analysis.

Microsoft Excel 2019 was used to create a trend chart of changes in the volume of documents issued each year, citations per article, total number of citations and H-index of top ten countries/regions and provide an intuitive development trend in this field.

We determine the importance of an article based on the assumption that highly cited articles may have a greater impact on the scientific literature. Co-citation means that two related articles (or authors) are cited by the third article (or author) at the same time. Articles with a common theme tend to form clusters around the same co-cited article pair, thereby highlighting their importance and relevance (Irmak, 2020).

VOSviewer was applied to analyze the co-citation of journals and authors. VOSviewer can give data of co-cited journals, authors, references and their citations, so that reflect the cooperative relationship and academic relations together with the visualization map created by CiteSpace.

We employed CiteSpace to visualize collaborations between countries/regions, organizations and authors and conduct co-citation analysis of authors, journals and references. In addition, we explored the changes in research directions and trends by creating a timeline view of co-cited reference. In order to better explore research hotspots, we used CiteSpace to capture keywords with strong citation bursts and constructed a visualization map.

## Result and Discussion

### Overall

**FIGURE 1:**
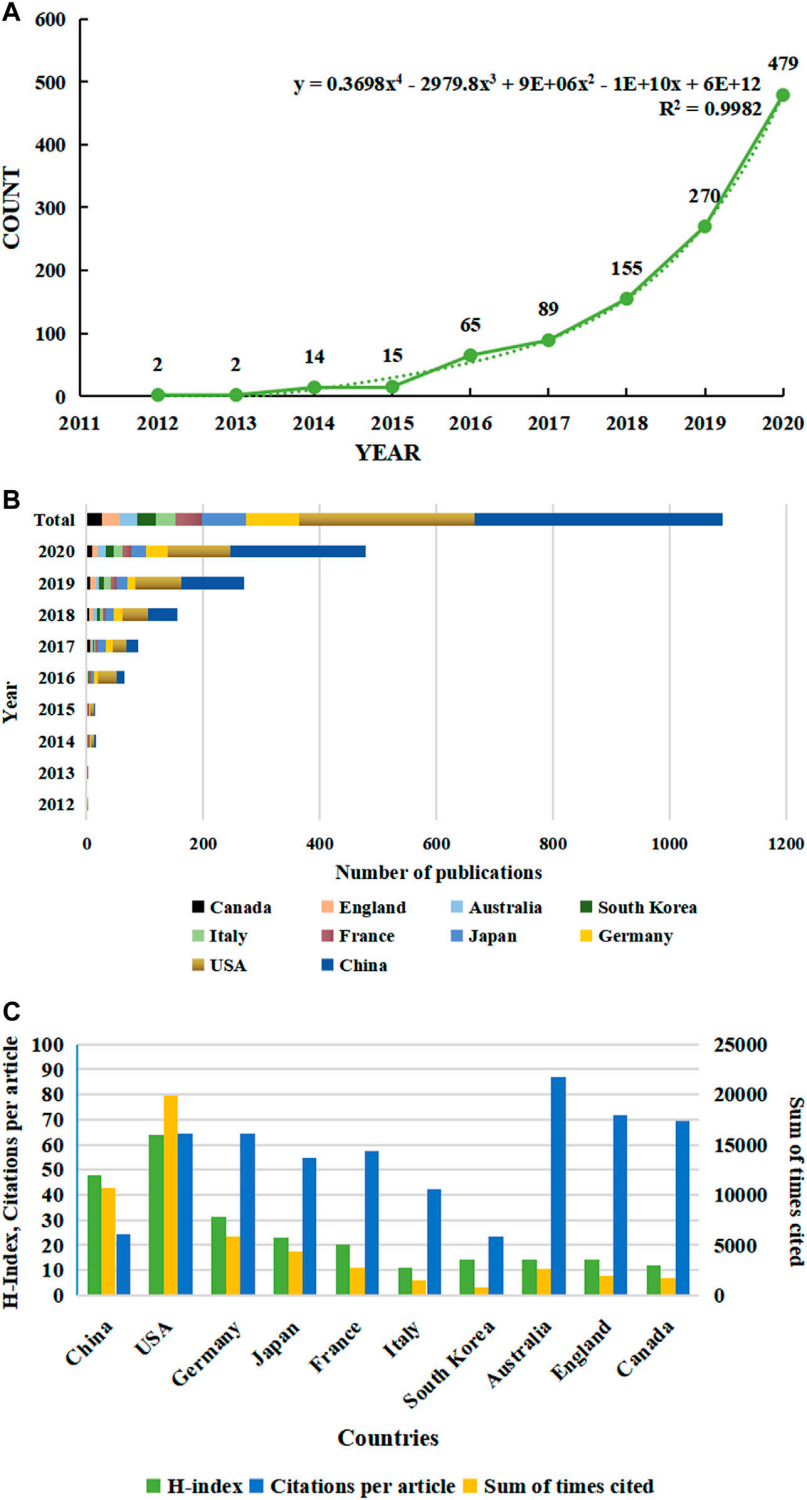
Trends in the number of publications and analysis of country/regions in ferroptosis in cancer research. **(A)** The annual worldwide publication output. **(B)** Growth trends in the publication output from the top 10 countries. **(C)** H-index, citations per article, and total number of citations for the top 10 country/regions.

In this article, we utilized information visualization to analyze original articles on ferroptosis from Web of Science (WOS) database from 2012 to 2020. A total of 965 publications met the search criteria. In the first 4 years (2012–2015) after the term ferroptosis was coined, the annual publication output maintained at a rather low level, but increased dramatically since 2016, and reached a peak in 2020 with the number of 550 (Figure1A). Through model fitting, we have obtained a growth curve of the volume of publications each year. Based on this curve, we conclude that ferroptosis is about to become an increasingly important subject.

It is worth noting that the annual publication output has even increased exponentially in the past 3 years. It reveals that the research of ferroptosis is developing rapidly, and attention has been increasing in the field of oncology, which is expected to become a hot issue in the future. The article published by Scott J. Dixon et al., in 2012 which confirmed that ferroptosis is a significantly different form of regulatory cell death has been cited the most frequently. This article is considered to be the most important and fundamental piece, which is pioneering research in this field (Dixon et al., 2012). Moreover, Xin Chen et al. published a review in Nature reviews, with the high IF (34.1060), on January 29, 2021. This review introduces the molecular mechanism of ferroptosis and tumor-related pathways, and explores the application of ferroptosis in cancer treatment, such as system therapy and immunotherapy. Therefore, more attention on ferroptosis will be triggered and more research on ferroptosis will be carried out (Chen et al., 2021).

### Country/Region and Institution Analysis

**FIGURE 2:**
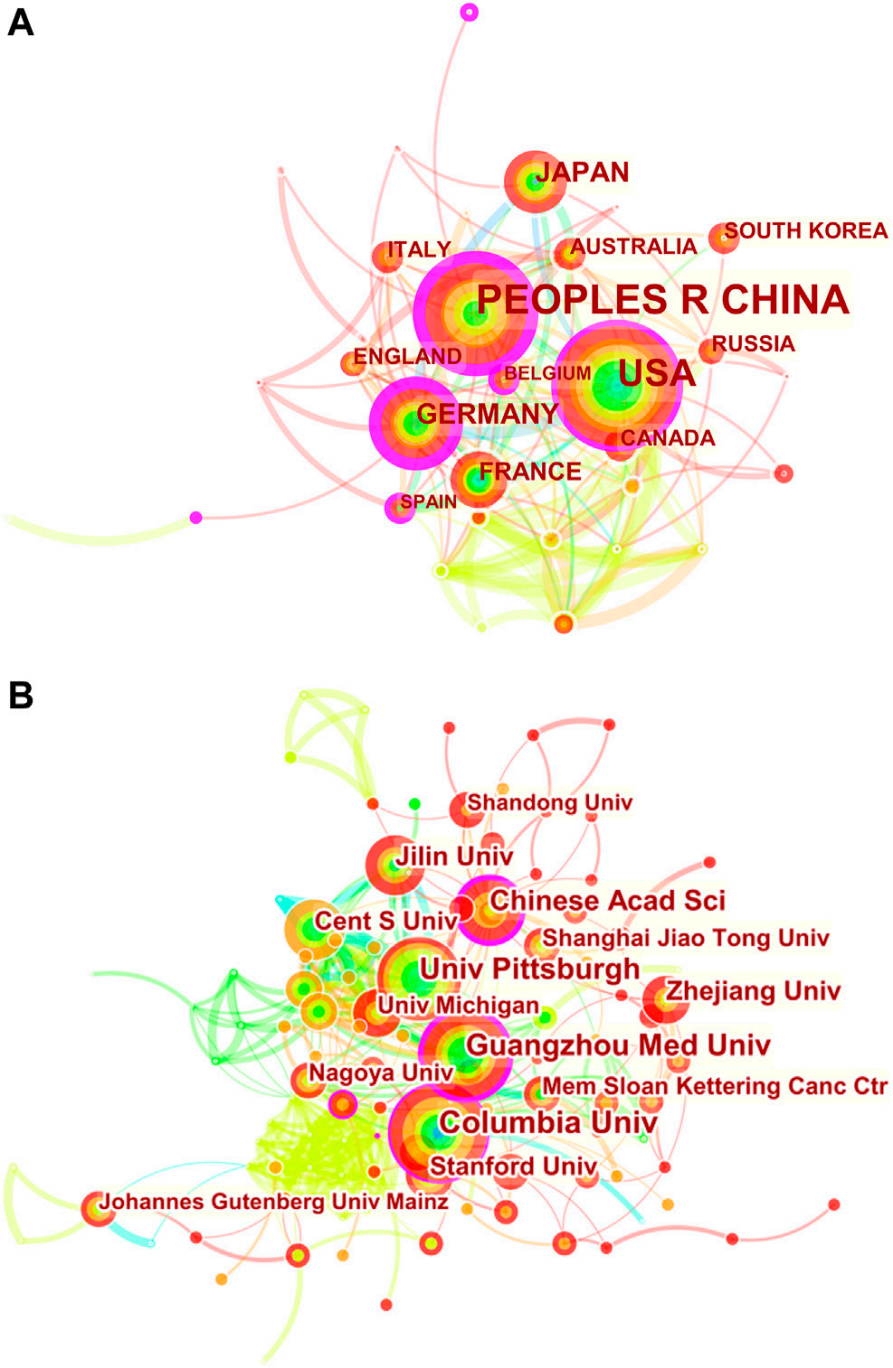
CiteSpace network visualization map of country/regions and institutions involved in ferroptosis in cancer research. **(A)** Collaboration analysis of countries/regions. **(B)** Collaboration analysis of institutions.

Publication related to ferroptosis was first published by American scholars in 2012, which was the only research article at that time. Their research is ahead of researchers in all regions of the world. All publications in the field were distributed among 1,169 institutions from 59 countries/regions, of which the production of People’s Republic of China ranked the first with 437 (45.28%) documents by far, followed by the United States (310, 32.12%), Germany (90, 9.33%), Japan (80, 8.29%) and France (47, 4.87%) (Table 1). Through the analysis of changes in the annual volume of documents issued of the 10 most productive countries/regions (Figure 1B), We can conclude that the overall trend in the annual number of documents published by those countries showed a similar increase from 2012. In horizontal comparison, the number of annual publications of China has been lower than that of the United States in the first 6 years since the term ‘ferroptosis’ was proposed in 2012, but the gap has gradually narrowed. And in 2018, the number of annual publications of China surpassed that of the United States and became the first. Although the total research output of countries, such as Germany, Japan, France, and Italy, is lower than that of China and the United States, there is also a significant growth in recent years. Obviously, one of the most important reasons is the close and friendly cooperation between different countries. Analysis of CiteSpace network visualization map of country/regions (Figure 2A) showed that there is a very strong cooperation between the United States and China, which has significantly impacted the research trend of ferroptosis.

**TABLE 1:** Top 10 productive country/regions related to ferroptosis in cancer research.

Rank	Countries/Regions	Articles (N)	Percentage (N/965)	H-index	Citations per article	Sum of times cited
1	China	437	45.28	48	24.51	10,712
2	United States	310	32.12	64	64.33	19,942
3	Germany	90	9.33	31	64.38	5,794
4	Japan	80	8.29	23	54.81	4,385
5	France	47	4.87	20	57.68	2,711
6	Italy	34	3.52	11	42.44	1,443
7	South Korea	32	3.32	14	23.19	742
8	Australia	30	3.11	14	87.00	2,610
9	Canada	26	2.69	14	71.96	1871
10	England	25	2.59	12	69.40	1735

When it comes to sum of times cited and citations per article, things are totally different (Figure1C and Table1). The USA had 19,942 citations and an H-index of 64, both of which ranked first among all included countries/regions, but its citation/article ratio (64.33) was lower than that of Canada (71.96), England (69.4) and Germany (64.38). As the most productive country/regions related to ferroptosis in cancer research, China had a relatively low citation/article ratio (24.51) when compared to the other countries in the table.

To explore international and inter-organizational cooperation, we constructed a network visualization map for publications on cancer research of ferroptosis by CiteSpace. Figure 2A shows collaborations among country/regions meeting the searching criterion (58). The countries marked with purple circles, including Germany (0.37), the United States (0.30) and People’s Republic of China (0.28) have strongest betweenness centrality, which means they played a key role in international collaboration. The connection between nodes represents the cooperative relationship between countries, and the width of the connection represents the strength of cooperation. Germany had the highest betweenness centrality, and the countries/regions that collaborated the most with Germany were Cameroon, Japan and Australia. Additionally, People’s Republic of China collaborated the most with the USA, Switzerland and France.

The 10 most productive institutions in relevant research are shown in Table 2. The leading institutions were University of Texas System (51,5.28%), Columbia University in the City of New York (44, 4.56%), Central South University (42,4.35%), Guangzhou Medical University (42, 4.35%) and Helmholtz Association (39, 4.04%). The collaborations among institutions are clearly shown in Figure 2B. Columbia University in the City of New York, Chinese Academy of Sciences, Central South University and University of Pittsburgh have strong betweenness centrality. Furthermore, it can be clearly seen that cooperation between American institutions is extremely active and close, with five institutions among the 10 institutions with the highest publication output. Regional advantages have been superlatively leveraged to a great extent, strengthening academic influence of the United States on this field.

**TABLE 2:** Top 10 productive institutions related to ferroptosis in cancer research.

Rank	Institutions	Articles (N)	Percentage (N/965)	Location
1	UNIV OF TEXAS SYSTEM	51	5.28	United States
2	COLUMBIA UNIV	44	4.56	United States
3	CENT S UNIV	42	4.35	China
3	GUANGZHOU MED UNIV	42	4.35	China
5	HELMHOLTZ ASSOCIATION	39	4.04	Germany
5	INSERM	39	4.04	France
7	PCSHE	38	3.94	United States
8	CHINESE ACAD SCI	35	3.63	China
8	UNIV OF PITTSBURGH	35	3.63	United States
10	UT SOUTHWESTERN	28	2.90	United States

### Journal Analysis

In total, 350 journals have contributed to the 965 publications. An additional file presents top 10 productive journals related to ferroptosis in cancer research [see Table 3], among which *Cell Death disease*, which had an IF of 6.304 in 2019, contains the most publications (30,3.11%). There is not much difference in the number of articles included in the top ten prolific journals, and five of the listed journals, namely *Cancer Research*, *Cancers*, *Cell Death and Differentiation*, *Oxidative Medicine and Cellular Longevity* and *Redox Biology* were tied for fifth place, contained 17 publications. *Cell Death and Differentiation* had the highest IF of 10.717 in 2019 among these ten journals, followed by *Redox Biology* (9.986). Regarding H-index, *Cancer Research* came first, but the documents it comprised ranked fifth. In the quartile category, 6 of the 10 journals were in Q1 (the top 25% of the IF distribution) of different areas, and the rest were in Q2 (25–50%). These results indicate that journals such as *Cell Death disease* have significant contributions in this field.

**TABLE 3:** Top 10 productive journals and co-cited journals related to ferroptosis in cancer research.

Rank	Journal	Count (N)	Percentage (N/965)	IF (2019)	H-index	Quartile in category
1	Cell Death Disease	30	3.11	6.304	85	Q1^d^
2	Biochemical and Biophysical Research Communications	28	2.90	2.985	243	Q3^a^
3	Free Radical Biology and Medicine	21	2.18	6.170	239	Q1^a^
4	International Journal of Molecular Sciences	18	1.87	4.556	80	Q2^c^
5	Cancer Research	17	1.76	9.727	411	Q1^b^
5	Cancers	17	1.76	6.126	53	Q1^b^
5	Cell Death and Differentiation	17	1.76	10.717	193	Q1^a^
5	Oxidative Medicine and Cellular Longevity	17	1.76	5.076	66	Q2^d^
5	Redox Biology	17	1.76	9.986	57	Q1^a^
10	Frontiers in Oncology	16	1.66	4.848	60	Q2^b^

a Biochemistry and molecular biology.

b Oncology.

c Multidisciplinary sciences.

d Cell biology.

The most co-cited journal is *Cell* (2,931 citations), with an IF of 38.637 in 2019 and an H-index of 705, followed by *Nature* (2,374 citations), *Journal of Biological Chemistry* (1738 citations), *Proceedings of the National Academy of Sciences of the United States* (1,675 citations) and *Cell Death and Differentiation* (1,338 citations). And all of the top 10 co-cited journals related to ferroptosis in cancer research were in Q1 except *Biochemical and Biophysical Research Communications* (Q3*).

### Author Analysis

**FIGURE 3:**
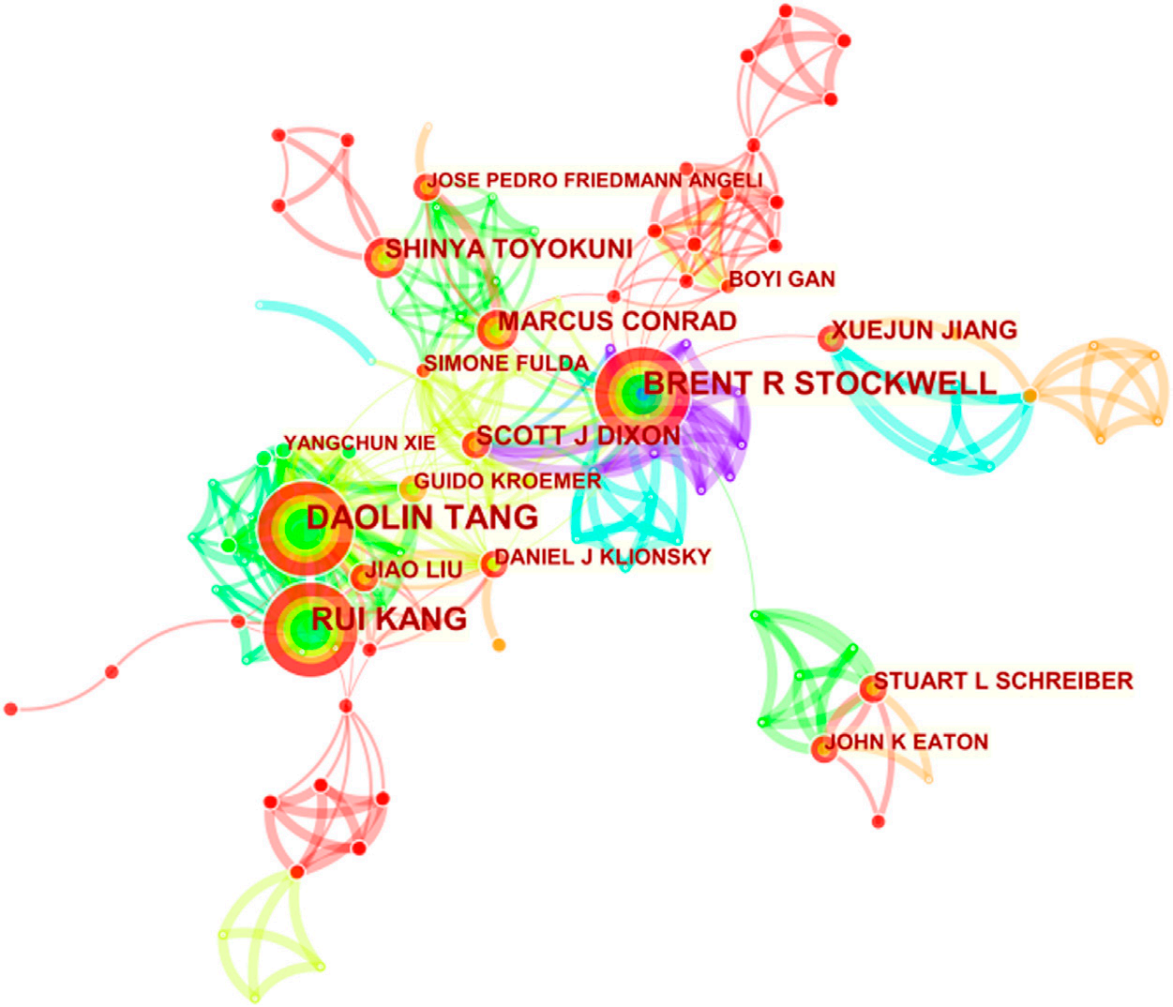
CiteSpace network visualization map of co-cited authors of the articles related to ferroptosis in cancer research.

The selected 965 publications were produced by 4,993 authors, and top 10 productive authors and co-cited authors are listed in Table 4. Tang DL headed with 38 documents, followed closely by Kang R with 37 documents. Stockwell BR and Dixon SJ are both the most productive and co-cited authors. The author’s co-cited network analysis was visualized in Figure 3. It is demonstrated in the figure that the most frequently co-cited author is Dixon SJ (1,188 citations), who is also the fifth most productive (17). Right after, Yang WS appears with 980 citations. Next are Gao MH (410 citations), Angeli JPF (385 citations) and Stockwell BR (359 citations). Among them, Dixon SJ (with 1,188 citations), Yang WS (with 980 citations) and Gao MH (with 410 citations) are all from the United States, of which the top two are from Columbia University, indicating that Columbia University produced high-quality articles in this field. However, most of the top 10 productive authors are from China but the frequency of citations is not as high as that of American scholars. It may be because China has only conducted a lot of research on ferroptosis over these years. Most of the articles were published in the past 3 years, and it takes time and more experiments to verify. This also shows the rapid development of ferroptosis in China in recent years, and Chinese scholars are enthusiastic about cancer research of ferroptosis.

**TABLE 4:** Top 10 productive authors and co-cited authors related to ferroptosis in cancer research.

Rank	Author	Count	Rank	Co-cited author	Citation
1	TANG DL	38	1	DIXON SJ	1,188
2	KANG R	37	2	YANG WS	980
3	STOCKWELL BR	32	3	GAO MH	410
4	LIU J	23	4	ANGELI JPF	385
5	TOYOKUNI S	20	5	STOCKWELL BR	359
6	CONRAD M	19	6	DOLL S	314
7	DIXON SJ	19	7	JIANG L	312
8	ZHANG Y	18	8	GALLUZZI L	292
9	LIU Y	15	9	XIE Y	254
9	WANG X	15	10	SUN XF	241

### Co-Cited References Analysis

**FIGURE 4:**
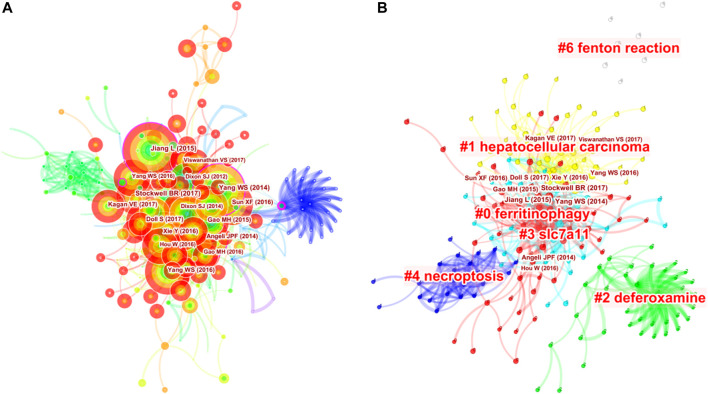
Analysis of references to ferroptosis in cancer research. **(A)** Network map of co-cited references. **(B)** Network map of co-cited clusters.

A total of 513 co-cited references were visualized by CiteSpace with the time slice set as a year, the time span set as 2012 to 2020 and the top 7% most cited selected (Figure 4A). Table 6 presents the top 10 cited references in ferroptosis in cancer treatment. Most of them are from the United States, and the first three are from the same institution--Columbia University. At the same time, Columbia University ranks highest in the list of contributing institutions. This shows that Columbia University attaches importance not only to the quantity of publications, but also to the quality of publications. Therefore, articles of Columbia University are worth reading and studying carefully. Most of the authors of the most cited articles are from one country, suggesting that experts on ferroptosis should be promoted and encouraged to communicate and cooperate with scholars from all over the world, so as to promote academic exchanges and joint cooperation. Nodes with high betweenness can be considered as pivotal points that provide important bridging connections between two research interests.

As shown in the additional file [see Table 5], the top 3 co-cited references were all published by Cell (IF 2019, 38.638 and H-index, 250), including the groundbreaking one authored by Dixon SJ (2012) that first proposed ferroptosis, who demonstrated erastin can trigger iron-dependent cell death--ferroptosis, which is regulated by a distinct set of genes, and put forward the concept of ferroptosis. Meanwhile, they proposed that non-apoptotic forms of cell death, such as ferroptosis, could promote selective death of tumor cells and protect the organism against neurodegeneration. The second is Yang WS al. (2014), which investigated the ability of ferroptosis inducers to inhibit the growth of tumor cells through GSH-dependent GPX4 inactivation in xenograft mouse models, demonstrating the importance of GPX4 in protecting cells from lipid peroxides. In addition, it concluded that diffuse large B cell lymphoma and renal cell carcinoma are specifically susceptible to ferroptosis by performing the erastin test on 117 different tissue cancer lines. Thus, GPX4, as the central regulatory factor of ferroptosis, can be regulated to induce ferroptosis in mouse tumor model, which provides a broad prospect for the therapeutic application of Ferroptosis inducing compounds.

**TABLE 5:** Top 10 co-cited references in ferroptosis in cancer research.

Rank	Co-citation	Centrality	Author	Title
1	679	0.06	Dixon SJ	Ferroptosis: An iron-dependent form of nonapoptotic cell death
2	463	0.18	Yang WS	Regulation of Ferroptosis Cancer Cell Death by GPX4
3	325	0.00	Stockwell BR	Ferroptosis: A Regulated Cell Death Nexus Linking Metabolism, Redox Biology, and Disease
4	264	0.12	Angeli JPF	Inactivation of the ferroptosis regulator Gpx4 triggers acute renal failure in mice
5	264	0.20	Jiang L	Ferroptosis as a p53-mediated activity during tumor suppression
6	245	0.01	Xie Y	Ferroptosis: Process and function
7	219	0.03	Dixon SJ	Pharmacological inhibition of cystine-glutamate exchange induces endoplasmic reticulum stress and ferroptosis
8	200	0.02	Doll S	ACSL4 dictates ferroptosis sensitivity by shaping cellular lipid composition
9	194	0.01	Yang WS	Ferroptosis: Death by Lipid Peroxidation
10	180	0.02	Gao MH	Glutaminolysis and Transferrin Regulate Ferroptosis

On this basis, we further constructed a network map to visualize the key clusters of co-cited references (Figure 4B). There were 6 clusters that met the criteria in all (Table 6). Cluster #0, namely ferritinophagy, was the largest cluster consisting of 63 references, followed by hepatocellular carcinoma (cluster #1), deferoxamine (cluster #2), SLC7A11 (cluster #3), necroptosis (cluster #4) and Fenton reaction (cluster #6).

**TABLE 6:** Top 10 largest clusters of co-cited references related to ferroptosis in cancer research.

Cluster ID	Size	Year	Top terms
#0	65	2016	Ferritinophagy
#1	47	2017	hepatocellular carcinoma
#2	39	2010	deferoxamine
#3	36	2013	slc7a11
#4	23	2013	necroptosis
#6	8	2017	Fenton reaction

**FIGURE 5:**
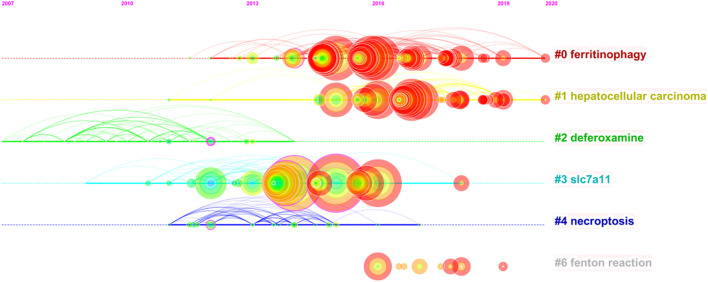
Timeline view of co-cited references related to ferroptosis in cancer research.

In order to figure out how research hotspots changed over time, we conducted a timeline view of co-cited references (Figure 5). Figure5 displays a timeline visualization of distinct co-citation and shows that Cluster #3 (SLC7A11) has the largest nodes scattered along the timeline and contains 6 of the 10 most frequently cited references, namely articles by Dixon SJ (Dixon et al., 2012; Dixon et al., 2014), Yang WS(Yang and Stockwell, 2016; Hangauer et al., 2017), Angeli JPF(Angeli et al., 2014), Jiang L (Jiang et al., 2015). In addition, it is obvious that the research on SLC7A11 started earlier, indicating that it has become mature theoretical research of ferroptosis mechanism (Lang et al., 2019). Meanwhile, it also implies that the occurrence mechanism of ferroptosis has not been clearly studied. Cluster #0 (ferritinophagy) and Cluster #1 (hepatocellular carcinoma) contains a large number of nodes with red rings, indicating that they were the latest research hotspots and directions of ferroptosis, which are worthy of attention. That is to say, the focus of the research seems to have shifted from the traditional mechanism to the treatment of hepatocellular carcinoma, which means that ferroptosis has a thorough and mature research on cancer, and can provide a new method of clinical treatment. Cluster #4 (necroptosis) and Cluster #6 (Fenton reaction) contain fewer nodes on the timeline, suggesting that this is a previous research hotspot or an emerging research direction. Therefore, future research should focus on comprehensive mechanism and clinical application, such as novel tumor therapies based on GPX4 covalent inhibitors, such as ML162 (Moosmayer et al., 2021). In addition, p53-regulated ferroptosis therapy or CD8^+^-mediated immunotherapy of cancer also has potential clinical value, which needs further research and mechanism elaboration (Chu et al., 2019; Kang et al., 2019; Stockwell and Jiang, 2019).

### Keyword Analysis

**FIGURE 6:**
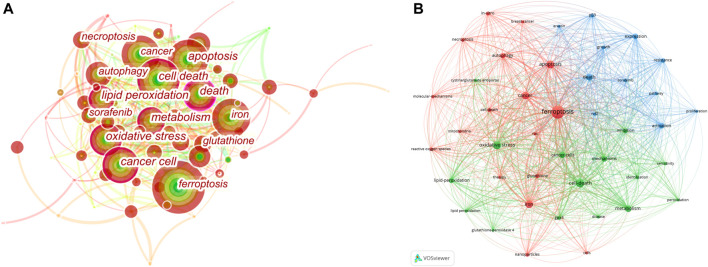
Analysis of Keyword Co-Occurrence. **(A)** CiteSpace network visualization map of co-occurring keywords. **(B)** VOSviewer network visualization map of co-occurring author keywords.

We used VOSviewer (Figure 6B) to analyze the keywords and formed three clusters, including terms related to mechanism, clinical application and molecular pathways. At the same time, in order to analyze the closeness of the relationship between different subjects, we used CiteSpace to create keyword co-occurrence map (Figure 6A). The size of nodes reflects frequency of keywords, and the width of the lines represents frequency of two keywords co-occurrence. The keywords with purple circle, such as ferroptosis, apoptosis and cell death, have strong betweenness centrality. It is obvious that ferroptosis mechanism has always been the hot spot of the research direction. Combined with the results of the burst keywords and timeline view of co-cited references, the research of ferroptosis in cancer therapy, especially hepatocellular carcinoma (HCC), is the currently main research direction. Sun et al. proved that the nuclear factor erythroid 2-related factor 2 (NRF2), as a key transcription regulator of ferroptosis, can protect HCC from ferroptosis. Moreover, ferroptosis-inducing compounds such as erastin and sorafenib, which is an emerging anti-cancer drug, can potentially enhance the anti-cancer activity by inhibiting the expression of NRF2. Therefore, NRF2 may become an effective target for the treatment of cancer through ferroptosis (Yang et al., 2014; Roh et al., 2017; Dodson et al., 2019).

Badgley et al. demonstrated that cysteine deficiency induces reduced glutathione (a cofactor of GPX4) synthesis, thereby inducing lipid peroxide-dependent iron metastases in mouse KRAS/p53 mutated pancreatic cancer (Badgley et al., 2020). System Xc-gene is the main pathway to obtain cysteine *in vivo*. The synthesis of small molecule Erastin, the knockout of SCL7A11, the main gene of this system, or the down-regulation of SCL7A11 caused by p53 upregulation can inhibit the system Xc-. SLC7A11 is overexpressed in many cancers, especially in pancreatic duct adenocarcinoma (PDAC), which is responsible for inhibition of ferroptosis on the one hand and resistance to ferroptosis on the other hand. Therefore, we speculated that cysteine removal therapy, SCL7A11 knockout or inhibition of expression, and system Xc-inhibition might be the pathways of PDAC targeted therapy. However, it has not been proven in human PDAC and is worthy of further study and investment. Studies have shown that NCoA4-mediated autophagy can degrade ferritin in cancer cells, thus inducing ferroptosis, which may be an effective anti-cancer treatment (Gao et al., 2016; Hou et al., 2016).

Wang et al. confirmed that ferroptosis can enhance the efficacy of cancer immunotherapy. CD8^+^ T cells down-regulate the expression of subunits (SCL3A2 and SCL7A11) in glutamate–cystine system Xc-by releasing INFγ to inhibit the uptake of cysteine in tumor cells, thereby inducing ferroptosis and contributing to cancer immunotherapy (Ubellacker et al., 2020). More and more studies have shown that the lymphatic system can promote the survival and metastasis of cancer cells, making it easier for cancers from lymph nodes to form metastatic tumors. The high level of oleic acid in the lymph fluid can inhibit the ferroptosis level of melanoma cells through the regulation of ASCL3, thereby increasing the metastasis of cancer cells. Therefore, we believe that enhancing the oxidative stress of cancer cells in the lymph and inducing iron death can reduce the survival rate of cancer cells during metastasis. It may become a new clinical treatment method to improve the prognosis of cancer (Liu et al., 2020; Tang et al., 2020).

### Burst Keywords Analysis

**FIGURE 7:**
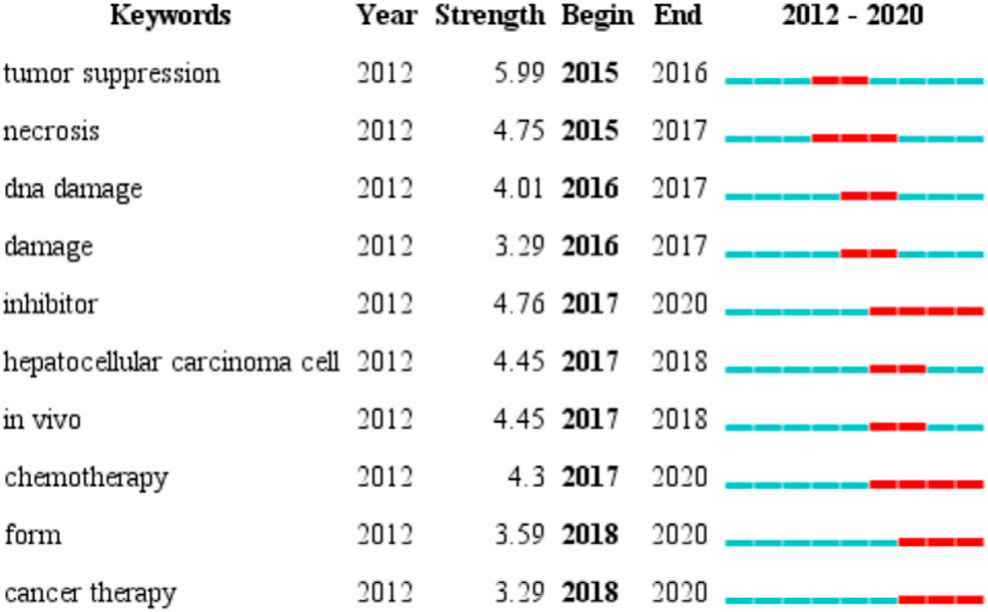
Top 10 burst keywords in articles related to ferroptosis in cancer research.

We used CiteSpace to detect top 10 keywords with the strongest citation bursts to provide helpful insights to research hotspots in this field (Figure 7). The strongest burst occurred in 2015, that tumor suppression had a burst strength of 5.99 and lasted 2 years, it indicates research on iron death in tumor suppression has begun to become a hot spot. The longest burst duration occurred in inhibitor (with a burst strength of 4.76), it started in 2017 and was still until we collected the data. Apart from the two keywords, we are particularly interested in those keywords that started to burst from 2018 onward, including form (with a burst strength of 3.59) and cancer therapy (with a burst strength of 3.29).

## Limitation

Compared with traditional reviews, CiteSpace-based analysis provides better insight into research focus and trends, but it also has certain limitations. First, this article does not include literature other than English literature, which may cause the source of bias. Additionally, the data used in this article was obtained only from the WoSCC database due to its reliability of the publications and citations. However, compared with other databases, this database has fewer literatures and journals, resulting in less comprehensive research results. Moreover, we selectively analyzed the characteristics of the information obtained in order to make the key points more prominent. Therefore, some details may be missed. Furthermore, some of the collaborative keywords shown in Figure 6 are similar and should be combined in the same circle, such as “death” and “cell death”, “cancer” and “cancer cell”. With the efforts of the CiteSpace research team, the software will continue to be updated to overcome this limitation and present more accurate and deeper knowledge in the future.

## Conclusion

In conclusion, our knowledge and understanding of ferroptosis has improved significantly through serious study of the previous high-quality articles. According to the search, our article is the first bibliometric analysis based on the results of CiteSpace and VOSviewer to study the research trends and hot spots of ferroptosis in cancer treatment. More and more studies have proved that ferroptosis can be applied to cancer treatment through the accumulation of lipid peroxidation. Besides, ferroptosis is also related to many diseases such as Parkinson’s disease, diabetic complications, renal failure, and cardiovascular and cerebrovascular diseases. From the detected burst of citations, we could find that the supplementary application of ferroptosis in chemotherapy is an emerging trend. With the assistance of information visualization, we are able to identify research priorities and general trends in the field, and make the collected information available to researchers in this field. Through further exploration of the mechanism of ferroptosis, therapeutic targets and new clinical treatment ideas are expected to be provided for more diseases.

## Abbreviations

H-index, Hirsch index; IF, Impact Factor; JCR, Journal Citation Reports; PDAC, Pancreatic duct adenocarcinoma; WOS, Web of Science; WoSCC, Web of Science Core Collection.
